# Anti-diabetic effects of palm fruit juice in the Nile rat (*Arvicanthis niloticus*)

**DOI:** 10.1017/jns.2014.3

**Published:** 2014-04-30

**Authors:** Julia Bolsinger, Andrzej Pronczuk, Ravigadevi Sambanthamurthi, K. C. Hayes

**Affiliations:** 1 Foster Biomedical Research Laboratory, Brandeis University, 415 South Street, Waltham, MA 02454, USA; 2 Malaysian Palm Oil Board, Kajang, Selangor, Malaysia

**Keywords:** Type 2 diabetes, Palm fruit juice, Oil palm phenolics, Nile rats, Insulin metabolism, Glucose metabolism, CHO, carbohydrate, GAE, gallic acid equivalents, OPP, oil palm phenolics, PFJ, palm fruit juice, T2DM, type 2 diabetes mellitus, TC, total cholesterol

## Abstract

With the increasing incidence of metabolic diseases, numerous bioactive phytochemicals have been proffered in the dietary prevention of these conditions. Palm fruit juice (PFJ) possesses bioactive phenolic compounds (referred to as oil palm phenolics; OPP) that may deter diabetes. The objective of the present experiments was to document the degree to which PFJ reduces diabetes symptoms in a variety of circumstances in the Nile rat (*Arvicanthis niloticus*), a novel model for carbohydrate-induced type 2 diabetes (type 2 diabetes mellitus; T2DM) and the metabolic syndrome. Wild-type male Nile rats (*n* 100) were fed laboratory chow or semi-purified diabetogenic diets in five experiments lasting 4–36 weeks. PFJ was provided as a drink or mixed into the diet to provide OPP intakes from 170 to 720 mg gallic acid equivalents/kg body weight per d. Body weight and random and fasting blood glucose were assessed at different time points, and were analysed along with terminal fasting organ weights, insulin, plasma and liver lipids as measures of diabetes progression. PFJ proved to be anti-hyperglycaemic and anti-lipaemic in all experiments relative to untreated controls, delaying T2DM onset and even reversing advancing diabetes. Protection by PFJ was directly related to its OPP content, and no negative effects on energy intake or growth were observed. PFJ was effective both as a drink and mixed into the diet. Results suggest that PFJ may slow the rate of glucose absorption, reduce insulin resistance and/or enhance insulin secretion.

With the increasing global incidence of type 2 diabetes mellitus (T2DM) and the metabolic syndrome, plant-derived bioactive phytochemicals are being introduced as a way to alleviate these metabolic disorders^(^^1^^)^. The anti-diabetogenic, cholesterol-lowering, anti-inflammatory and other metabolically beneficial qualities of phytochemicals from various plant extracts have been evaluated in a number of animal and human studies^(^^2^^–^^4^^)^. Palm fruit juice (PFJ) is a water-soluble by-product of palm oil extraction from the fruit of the oil palm (*Elaeis guineensis*), and is a rich source of such phytochemicals, particularly bioactive oil palm phenolics (OPP)^(^^5^^)^. A PFJ extract containing OPP was found to have anti-diabetogenic qualities in preliminary studies^(^^6^^,^^7^^)^, but the preferred concentration of OPP, its optimal dosage and delivery form as food or drink, as well as the mechanism by which OPP modulates blood glucose, insulin and blood lipids in the context of the metabolic syndrome are yet to be determined.

It has been appreciated for decades that polyphenols, for example from extracts of fruit or rhisomes, or certain legumes such as beans and lentils, reduce glucose absorption and benefit animals and humans afflicted with T2DM^(^^8^^–^^12^^)^. Accordingly, we explored a cost-effective source of polyphenols for possible use in such individuals, including the expanding worldwide population of pre-diabetics. Initial studies with PFJ are described here using the Nile rat (*Arvicanthis niloticus*), a novel, carbohydrate (CHO)-sensitive rodent model for T2DM and the metabolic syndrome. It spontaneously develops hyperglycaemia with insulin resistance and β-cell failure, as well as other symptoms of the metabolic syndrome, including hyperlipaemia with elevated TAG and depressed HDL-cholesterol, and elevated blood pressure^(^^13^^–^^15^^)^. The onset and severity of its symptoms can be modified by dietary intervention, with a high intake of simple CHO leading to the most severe symptoms^(^^15^^)^. The present experiments examined different concentrations and dosage forms of PFJ on the development of CHO-induced diabetes in male Nile rats.

## Methods

### Animals and diets

A total of 100 male Nile rats were tested in five different experiments in order to gain an extensive overview of the effects of PFJ on diabetes in this rodent model. Starting ages of 3 weeks and 12 weeks were evaluated, and intervention periods ranged from 4 weeks to 36 weeks. PFJ was provided as a drink or mixed into semi-purified diets in which the macronutrient composition was specifically designed to induce diabetes in this model. In all experiments except experiment 5 (high-CHO diet rich in dextrose), an energy-free sweetener (Splenda^®^ TM; McNeil Nutritionals LLC; 1 g/l) was added to the PFJ to compensate for its natural bitterness. The composition of PFJ was as described previously^(^^16^^)^, with total solids composed mainly of CHO (65 %, mainly sucrose and fibre), protein (12 %), ash (20 %) and OPP (about 3·5 %). For any selected extract the phenolic content (OPP) was measured as gallic acid equivalents (GAE) by spectrophotometric assay^(^^17^^)^. Food and water intake, body weight, random and fasting blood glucose, OPP intake, terminal organ weights as well as plasma lipids and insulin were assessed. All experiments and procedures were approved by the Brandeis University Institutional Animal Care and Use Committee.

### Experiment 1

To extend our preliminary observation on the anti-diabetogenic qualities of PFJ, as well as its possible negative long-term effects on growth or metabolism, sixteen male wild-type Nile rats received either water or PFJ containing 1500 mg/l GAE (eight rats per group), while consuming standard laboratory chow *ad libitum* (LabDiet^®^ no. 5020; CHO–fat–protein = 57:21:22 % energy, 15·9 kJ/g). Starting age was 12 weeks, and total study duration was 36 weeks. Food and drink intakes were recorded (between 0 and 4 weeks and again between 33 and 36 weeks of intervention), and bottles were exchanged three times per week. Body weight and fasting blood glucose (16 h overnight fast) were recorded initially and again after 12 and 36 weeks. Random blood glucose was also assessed terminally (after 36 weeks). Rats were subsequently fasted overnight and exsanguinated via cardiac puncture under 50:50 O_2_–CO_2_ anaesthesia. Plasma samples were frozen immediately for lipid and insulin analyses. Organs were removed and weighed.

### Experiment 2

To assess the relationship between phenolic content of PFJ and its anti-diabetogenic effect, a PFJ concentrate was diluted in water to supply various concentrations of OPP (at 0, 450, 900 and 1800 mg/l GAE) as a drink for twenty-seven male wild-type Nile rats (six or seven rats per group). Starting age was 12 weeks, and the intervention period was 17 weeks. Rats again were fed standard laboratory chow (LabDiet® no. 5020) *ad libitum*. Food and drink intakes were recorded (drink intake was recorded for the first 4 weeks and from 9 to 12 weeks). Body weight and fasting blood glucose (16 h overnight fast) were recorded after 9 and 17 weeks, at which time rats were fasted for 16 h and exsanguinated via cardiac puncture under a 50:50 O_2_–CO_2_ anaesthesia. Plasma samples and organs were collected as described in experiment 1.

### Experiment 3

To assess the effectiveness of providing PFJ blended directly in a semi-purified diet, experiment 3 fed a moderate-CHO, moderate-fat diet (CHO–fat–protein = 40:43:17 % energy, 18·8 kJ/g) (Table 1) as the diabetogenic control diet. Experiment 3 used normoglycaemic rats in a long-term prevention trial of 24 weeks with PFJ (13 000 mg/l GAE) added as 415 ml/kg dry diet to provide a final concentration of 5·4 g GAE/kg diet. A total of twenty-three 8-week-old normoglycaemic (mean random blood glucose 3·5 mmol/l) male wild-type Nile rats (eleven or twelve rats per group) were fed their respective diets *ad libitum* (Table 4). Body weight and random blood glucose were assessed after 24 weeks, and rats were subsequently necropsied as described for experiments 1 and 2.

**Table 1.** Diet composition for all experiments

Diet...	Moderate-CHO	High-CHO	Chow (LabDiet^®^ 5020)*
Energy percentage			
Carbohydrate	40	70	57
Fat	43	10	21
Protein	17	20	22
Energy (kJ/g)	18·8	16·7	15·9
Fed in experiment	3 and 4	5	1 and 2
Ingredients (g/kg)			
Casein	80	100	
Lactalbumin	80	100	
Oatmeal (7·5 % fat)	200	0	
Dextrose	170	350	
Maize starch	110 (+60 with gel)†	288 (+60 with gel)†	
Cellulose	31	0	
Fibre from oatmeal	20	0	
Fat	198	44	
Butter (amount of fat)	40	8	
Tallow	97	15	
Lard	31	0	
Soyabean oil	30	23	
Mineral mix‡	52	44	
Vitamin mix§	14	11	
Choline chloride	3	3	
Cholesterol	0·6‖	0·6	

CHO, carbohydrate; PFJ, palm fruit juice; ppm, parts per million; GAE, gallic acid equivalents.

* Main ingredients: ground wheat, ground maize, dehulled soyabean meal and wheat germ.

† 60 g maize starch were added to 800 ml water to form a gel, or added to 375 ml water + 415 ml PFJ (13 000 ppm GAE, experiments 3 and 5), or added to 800 ml PFJ (13 000 ppm GAE, experiment 4).

‡ Ausman-Hayes salt mix. Mineral mix contained the following (g/kg mix): magnesium oxide, 320; calcium carbonate, 290·5; potassium phosphate dibasic, 312·2; calcium phosphate dibasic, 72·6; magnesium sulfate, 98·7; sodium chloride, 162·4; ferric citrate, 26·6; potassium iodide, 0·77; manganese sulfate, 3·66; zinc chloride, 0·24; cupric sulfate, 0·29; chromium acetate, 0·044; sodium selenite, 0·004.

§ Hayes-Cathcart vitamin mix. Vitamin mix contained the following (g/kg mix): d-α-tocopheryl acetate (500 IU/g), 15; inositol, 5; niacin, 3; calcium pantothenate, 1·6; retinyl palmitate (500 000 IU/g), 1·5; cholecalciferol (400 000 IU/g), 0·100; menadione, 0·200; biotin, 0·020; folic acid, 0·200; riboflavin, 0·700; thiamin, 0·600; pyridoxine HCl, 0·700; cyanocobalamin, 0·001; dextrin, 972.

‖ Including 0·2 g cholesterol from butter, tallow and lard.

### Experiment 4

To test the possibility of reversing diabetes with PFJ, ten 12-week-old males with pre-existing random hyperglycaemia (mean random blood glucose about 17·0 mmol/l; Table 5) were fed a moderate-CHO diet with a PFJ concentration roughly doubled compared with that in experiment 3. Thus, 800 ml of the same PFJ concentrate (13 000 mg/l GAE) were added directly to the diet to provide a concentration of 10·4 g GAE/kg dry diet. Rats were divided into two groups (five rats per group) and the intervention time was reduced to 6 weeks, reflecting the greater intake of PFJ and OPP. Food, drink and body weight were followed. Since a more rapid response was expected with the increased concentration of OPP in experiment 4, rats were weighed and random blood glucose was measured after 6 weeks of intervention, when the study was terminated and tissues collected as described above.

### Experiment 5

To compare application method (PFJ as a drink *v.* mixed into the diet) within the same study, experiment 5 fed a semi-purified high-CHO diet (CHO–fat–protein = 70:10:20 % energy, 16·7 kJ/g) (Table 1) to twenty-three male wild-type Nile rats distributed among three groups (seven or eight per group). Since results from other experiments^(^^13^^)^ had shown that a younger starting age leads to more rapid diabetes induction, 3-week-old male wild-type rats were studied for 4 weeks. Control rats consumed the diabetogenic high-CHO diet and received water, and the two groups of intervention rats received the same diet plus PFJ as a drink (1500 mg/l GAE), or the high-CHO diet with 415 ml PFJ (13 000 mg/l GAE) incorporated directly into the diet (Table 1). Body weight, fasting and random blood glucose were assessed after 4 weeks, and rats were exsanguinated and necropsied as described above.

### Organ weight

Organs were weighed after excision, and their weight (in g) was divided by terminal body weight (in g). The relative carcass weight (as percentage body mass) was determined by weighing lean body mass (after exsanguination and excision of all organs) and dividing it by terminal body weight. Carcass weight was included as an indicator of muscle growth (mass).

### Blood glucose

Blood glucose was measured in O_2_–CO_2_-anaesthetised rats from a drop of tail blood, obtained by lancet puncture of the lateral tail vein using an Elite XL glucometer (Bayer Co.). Random blood glucose was assessed in non-fasted rats at about 09.00–10.00 hours on non-feeding days (semi-purified diets provided three times per week). Fasting blood glucose was measured at about 09.00–10.00 hours after 16 h overnight food deprivation. Based on experience from the initial experiments (experiments 1 and 2) and data detailed previously^(^^15^^)^, random blood glucose was identified as an early and more reliable parameter of diabetes in the Nile rat than fasting blood glucose. Thus, in the later experiments (experiments 3, 4 and 5), random blood glucose was used to assess the disease stages, which allowed for shorter intervention periods. A more detailed rationale for using random blood glucose as a diabetes assessment parameter in the Nile rat can be found in a previously published paper^(^^15^^)^.

### Plasma TAG and total cholesterol

Plasma TAG and total cholesterol (TC) were determined spectrophotometrically using Infinity^TM^ kits (TAG ref no. TR22421, TC ref no. TR13421; Thermo Fisher Scientific Inc.). The precision of the TAG assay is 1·66 % for intra-assay variation and 3·35 % for inter-assay variation. The precision of the TC assay is 2·15 % for intra-assay variation and 2·15 % for inter-assay variation.

### Liver lipids

Liver TAG and TC were extracted from 0·1 g of tissue ground with 4 g of sodium sulfate using a 2:1 chloroform–methanol solution. Total extract was combined and dried under N_2_ and re-dissolved in 1 ml of chloroform. A sample (10–20 µl) of each sample was dried under N_2_ and dissolved in 50 µl of Triton X-100 and chloroform (1:1, v/v). The solution was dried extensively to remove chloroform, and TAG and TC were determined using the appropriate Infinity^TM^ kit (Thermo Fisher Scientific Inc.). The precision of the liver TAG assay is 2·56 % for intra-assay variation and 5·51 % for inter-assay variation. The precision of the liver TC assay is 4·04 % for intra-assay variation and 4·56 % for inter-assay variation.

### Insulin ELISA for insulin

Plasma insulin was determined with an ELISA kit for rat/mouse insulin (catalogue no. EZRMI-13 K; Linco Research, Millipore), according to the manufacturer's protocol. The precision of this plasma insulin assay is 1·91 % for intra-assay variation and 7·63 % for inter-assay variation.

### Food efficiency

Food efficiency was calculated by dividing body-weight gain (g/d) by energy intake (daily food intake in kJ/d) and multiplying the result by 1000. Results are presented as g body weight gained per 1000 kJ consumed. Thus, greater food efficiency represents greater weight gained per kJ.

### Statistical analysis

Statistical analysis was performed using Super ANOVA statistical software (Abacus Concepts, Inc.). Student's *t* tests, one-way ANOVA, paired *t* tests or repeated-measures ANOVA with a *post hoc* Fisher's protected least significant difference (PLSD) test were conducted where appropriate to the study design. All data were checked for normality and, if not normally distributed, were normalised before statistical analysis through logarithmic, arcsin or arctan conversion. A *P* value of < 0·05 was considered statistically significant.

## Results

All studies revealed protective characteristics of PFJ against hyperglycaemia and hyperlipaemia, with a clear relationship between OPP intake and the anti-diabetic effect. Control rats were more susceptible to the diet-induced diabetes when challenged with a high-CHO diet at a younger age (experiment 5), which is consistent with our previous studies^(^^13^^,^^15^^)^. PFJ protection against blood glucose elevation was evident independent of type of diet (chow or semi-purified), starting age, study duration, initial blood glucose, or application method (PFJ added to the diet or provided as a drink).

### Experiment 1: palm fruit juice (1500 mg/l gallic acid equivalents) as a drink with chow

#### Energy intake, growth and blood glucose

The relatively mature 12-week-old rats in the control and PFJ groups in experiment 1 gained comparable weight (30–36 g) during the 36-week study (Table 2). Energy intake from food was significantly greater for control rats developing hyperphagia with diabetes onset, but after accounting for sugar energy in PFJ in the drink group, total energy intake did not differ between groups. A tendency still existed for PFJ rats to consume less energy, and their food efficiency was significantly greater than that of controls. In addition, control rats increased their fasting blood glucose significantly (*P* < 0·05) from baseline and had higher fasting blood glucose than PFJ rats (*P* < 0·05) after 36 weeks of feeding, as well as higher terminal random blood glucose. Interestingly, random blood glucose in PFJ rats also became modestly elevated after 36 weeks. In addition, in accordance with previous studies^(^^13^^,^^15^^)^, in both groups the random blood glucose was reduced approximately 50 % by overnight fasting.

#### Organ weights and plasma parameters

Terminal liver, kidney and caecum were significantly enlarged in control rats, in accord with their advanced diabetes (Table 2). Fasting plasma TC and TAG were also significantly elevated in the control group in keeping with advancing metabolic syndrome, while PFJ rats revealed only mild plasma lipid increases. There was no significant difference in terminal plasma insulin between the two groups, and insulin was about 50 % higher in PFJ rats than controls.

**Table 2.** Effect of palm fruit juice (PFJ)† on onset of diabetes in 12-week-old male Nile rats (*Arvicanthis niloticus*) fed chow‡ for 36 weeks (experiment 1) (Mean values and standard deviations)§

Group...	Water		PFJ 1500 drink	
	Mean	sd	Mean	sd
Rats (*n*)	8		8	
BW (g)				
Initial at age 12 weeks	94	14	95	15
After 36 weeks	124	12	131	17
Food intake				
g/d, 0–4 weeks	16	5	10*	2
g/d, 33–36 weeks	20	5	11*	1
kJ/d, 0–4 weeks	251	79	159*	33
kJ/d, 33–36 weeks	314	79	172*	17
Drink intake‖				
ml/d, 0–4 weeks	32	19	42	5
ml/d, 33–36 weeks	68	25	54	6
kJ/d, 0–4 weeks	0		25	4
kJ/d, 33–36 weeks	0		29	12
Total energy intake (kJ/d)	285	79	197	17
Food efficiency (g BW gained/1000 kJ)	0·5	0·1	0·8*	0·1
GAE intake (mg/kg BW per d)	0		648	
Random blood glucose, after 36 weeks (mmol/l)	22·3	7·7	11·1*	5·6
Fasting blood glucose (mmol/l)				
Initial	3·0^a,b^	0·8	2·9	1·4
After 12 weeks	6·8^a,c^	5·3	3·4	1·8
After 36 weeks	10·6^b,c^	6·0	4·3*	3·2
Organ weight (% BW)				
Liver	6·3	1·3	4·6*	0·9
Kidney	1·3	0·3	1·0*	0·1
Caecum	2·7	1·1	1·7*	0·5
Adipose				
Epididymal	2·6	1·0	2·8	0·5
Perirenal	0·8	0·4	1·3	0·5
Inguinal	0·8	0·1	1·0*	0·2
Omental	0·9	0·2	0·9	0·2
Brown	1·5	0·7	1·8	0·5
Total adipose	6·7	1·7	7·8	1·5
Carcass	67	3	70	2
Fasting plasma lipids, 36 weeks (mmol/l)				
TC	9·5	4·1	5·8*	2·6
TAG	6·6	7·0	1·3*	1·0
Fasting plasma insulin, 36 weeks (pmol/l)	0·9	0·6	1·4	0·3

BW, body weight; GAE, gallic acid equivalents; TC, total cholesterol; ppm, parts per million; CHO, carbohydrate.

^a,b,c^ Mean values within a column sharing a common superscript were significantly different (*P* < 0·05; repeated-measures ANOVA and Fisher's protected least significant difference (PLSD) test).

* Mean value was significantly different from that of the water group (*P* < 0·05; unpaired *t* test).

† Drink of PFJ containing 1500 ppm GAE.

‡ Laboratory chow 5020, percentage energy from CHO–fat–protein = 57:21:22, 15·9 kJ/g.

§ Data normalised by log transformation for statistical analysis as necessary.

‖ Values include energy from PFJ sugars.

### Experiment 2: graded intakes of palm fruit juice in water (0, 450, 900 and 1800 mg/l gallic acid equivalents)

#### Energy intake, growth and blood glucose

In experiment 2, all four groups gained weight normally (about 20 g) during the 17-week intervention (Table 3). Terminal body weights did not differ between the groups, but the control group (water with 0 mg/l GAE) had the lowest overall weight gain, even though their total energy intake was greater than that of PFJ rats (including adjustment for energy from PFJ sugars). Similarly to experiment 1, food efficiency was thus reduced in the control group compared with all three groups supplemented with PFJ. Fasting blood glucose was significantly elevated in the control group compared with the two groups consuming the most concentrated PFJ after 9 and 17 weeks, with fasting blood glucose in rats receiving the lowest intake of PFJ being intermediate. Thus, fasting blood glucose was inversely correlated with OPP intake (as mg GAE/kg body weight per d) (*r* −0·94; *P* < 0·001), with the most pronounced hyperglycaemia observed in control rats and the lowest (normal) blood glucose in rats consuming PFJ at 1800 mg/l GAE.

**Table 3.** Dose-dependent protective effects of graded intakes of palm fruit juice (PFJ)* against diabetes in 12-week old male Nile rats (*Arvicanthis niloticus*) fed chow† for 17 weeks (experiment 2) (Mean values and standard deviations)‡

Group...	Water		PFJ 450 drink		PFJ 900 drink		PFJ 1800 drink	
	Mean	sd	Mean	sd	Mean	sd	Mean	sd
Rats (*n*)	6		7		7		7	
BW (g)								
Initial at age 12 weeks	95	20	98	24	95	14	98	12
After 9 weeks	108	18	116	22	111	19	110	15
After 17 weeks	111	18	122	22	117	18	118	20
Food intake								
g/d	18^a,b^	5	14	4	12^a^	2	12^b^	3
kJ/d	276^a,b^	75	226	67	192^a^	29	184^b^	42
Drink intake§								
ml/d, 0–4 weeks	26^a,b,c^	8	34^a^	12	40^b^	13	43^c^	6
ml/d, 9–12 weeks	66	34	50	21	44	16	32	17
kJ/d, 9–12 weeks	0		8	4	13	4	21	8
Total energy intake (kJ/d)	276^a,b^	75	234	67	205^a^	29	205^b^	42
Food efficiency (g BW gained/1000 kJ)	0·5^a,b,c^	0·1	0·9^a^	0·2	0·9^b^	0·1	0·8^c^	0·2
GAE intake (mg/kg BW per d)	0		170		360		627	
Fasting blood glucose (mmol/l)								
Initial	2·7	1·3	3·8	2·2	3·7	2·4	3·8	1·3
After 9 weeks	9·7^a,b,c^	8·1	4·4^a,d^	2·2	4·3^b,e^	3·4	1·9^c,d,e^	0·3
After 17 weeks	7·7^a,b^	6·1	5·3^c^	2·8	4·1^a^	2·6	2·7^b,c^	0·9
Organ weight (% BW)								
Liver	5·4^a,b^	1·4	4·5	0·9	4·3^a^	1·0	3·8^b^	0·6
Kidney	1·4^a,b,c^	0·7	1·0^a^	0·2	0·9^b^	0·2	0·9^c^	0·2
Caecum	2·0	1·4	2·0	0·4	1·5	0·4	1·7	1·1
Adipose								
Epididymal	2·5	0·7	2·4	1·1	3·0	0·9	2·9	1·3
Perirenal	0·8	0·4	0·8	0·6	1·3	0·5	1·3	0·6
Brown	1·3	0·9	1·6	1·4	1·7	0·6	2·0	0·8
Total adipose	6·3	1·0	6·6	3·3	7·8	2·0	8·3	3·0
Pancreas	0·5	0·1	0·5^a,b^	0·0	0·6^a^	0·2	0·6^b^	0·1
Carcass	68	2	66	8	70	3	70	1
Fasting liver lipids, 17 weeks (mg/g)								
TC	21	9	18	9	22	9	24	11
TAG	51^a,b^	33	53	17	84^a^	24	82^b^	33
Fasting plasma lipids, 17 weeks (mmol/l)								
TC	10·5^a,b,c^	6·2	5·1^a^	0·9	4·1^b^	1·0	4·5^c^	2·1
TAG	2·5^a,b^	2·1	1·3	0·8	1·0^a^	0·5	0·7^b^	0·3
Fasting plasma insulin, 17 weeks (pmol/l)	0·5^a^	0·2	0·2^b,c^	0·1	0·8^b^	0·2	0·9^a,c^	0·3

BW, body weight; GAE, gallic acid equivalents; TC, total cholesterol; ppm, parts per million; CHO, carbohydrate.

^a,b,c,d,e^ Means within a row sharing a common superscript were significantly different (*P* < 0·05; one-way ANOVA and Fisher's protected least significant difference (PLSD) test).

* Drink of PFJ containing 450, 900 or 1800 ppm GAE.

† Laboratory chow 5020, percentage energy from CHO–fat–protein = 57:21:22, 15·9 kJ/g.

‡ Data normalised by log transformation for statistical analysis as necessary.

§ Values include energy from PFJ sugars.

#### Organ weights, plasma and liver parameters

As in experiment 1, terminal liver and kidney weights were significantly greater in control rats, reflecting their advanced diabetes (Table 3). The pancreas weighed less in both the control (no PFJ) and PFJ at 450 mg/l GAE groups compared with the two higher PFJ intake groups (900 and 1800 mg/l GAE). The pancreas mass also directly reflected the terminal fasting plasma insulin, which was lower in the 450 mg/l GAE group compared with control rats (NS). However, insulin was significantly higher for the 900 and 1800 mg/l GAE groups. Fasting plasma TC and TAG were significantly increased in the control group compared with PFJ groups (typical TC in the Nile rat about 4·5 mmol/l). No differences were observed in terminal liver TC, but liver TAG was greatest (about 83 mg/g) in the two groups with the highest OPP intakes compared with the control group and that with the lowest PFJ intake (*P* < 0·05).

### Experiment 3: prevention of diabetes by palm fruit juice mixed into semi-purified diet

#### Energy intake, growth and blood glucose

Experiment 3 examined long-term prevention with PFJ in a semi-purified diet at 5·4 g GAE/kg of diet. This supplied PFJ at 400 mg/kg body weight per d without resulting in differences in body weight or energy intake between control and test groups at any point over the 24 weeks (Table 4). Random blood glucose was significantly elevated in control rats relative to the PFJ group by 24 weeks. A similar difference was noted for terminal fasting blood glucose. It is noteworthy that both groups increased their random blood glucose significantly from baseline after 24 weeks (*P* < 0·05).

**Table 4.** Effect of palm fruit juice (PFJ)‡ on onset of diabetes in 8-week-old male Nile rats (*Arvicanthis niloticus*) when added to a semi-purified moderate-carbohydrate (CHO) diet§ for 24 weeks (experiment 3) (Mean values and standard deviations)‖

Group...	Moderate-CHO		Moderate-CHO + PFJ	
	Mean	sd	Mean	sd
Rats (*n*)	11		12	
BW (g)				
Initial at 8 weeks of age	70	10	70	6
After 24 weeks	122	12	115	12
Food intake				
g/d	8	1	7	1
kJ/d	146	13	142	13
Water intake				
ml/d, 0–20 weeks	10	5	7	2
ml/d, 20–24 weeks	25	16	11*	7
Food efficiency (g BW gained/1000 kJ)	1·9	0·3	1·9	0·2
GAE intake (mg/kg BW per d)	0		409	
Random blood glucose (mmol/l)				
Initial at 8 weeks of age	3·6	1·1	3·4	0·8
After 24 weeks	16·2†	7·9	7·5*†	5·8
Fasting blood glucose (mmol/l)				
After 24 weeks	11·7	10·7	5·1*	3·5
Organ weight (% BW)				
Liver	4·4	1·2	3·9	1·0
Kidney	1·0	0·3	0·8*	0·0
Caecum	1·5	0·4	1·4	0·3
Adipose				
Epididymal	3·4	0·7	3·7	0·9
Perirenal	1·9	0·9	1·9	0·8
Brown	2·1	0·7	2·3	0·9
Total adipose	7·3	1·5	7·9	1·5
Carcass	75	4	75	3
Fasting liver lipids, 24 weeks (mg/g)				
TC	74	29	71	17
TAG	24	7	29	10
Fasting plasma lipids, 24 weeks (mmol/l)				
TC	6·0	2·9	4·1*	1·0
TAG	4·4	3·8	2·0*	0·1

BW, body weight; GAE, gallic acid equivalents; TC, total cholesterol; ppm, parts per million.

* Mean value was significantly different from that of the moderate-CHO group (*P*<0·05; unpaired *t* test).

† Mean value was significantly different from that at 8 weeks of age (*P* < 0·05; paired *t* test).

‡ 415 ml of PFJ 13 000 ppm GAE for final concentration of 5·4 g GAE/kg diet.

§ Percentage energy from CHO–fat–protein = 40:43:17, 18·8 kJ/g.

‖ Data normalised by log transformation for statistical analysis as necessary.

#### Organ weights, plasma and liver lipids

Kidney (*P* < 0·05) and liver weights were greater for control rats, in accordance with the observations from experiments 1 and 2. Both fasting plasma TC and TAG were significantly lower for the PFJ group than for control rats.

### Experiment 4: reversal of diabetes by palm fruit juice in diet

In experiment 4, body weight did not differ significantly between groups at any point in the experiment (Table 5), but controls consumed significantly more energy throughout the 6-week test period, resulting in higher food efficiency for the PFJ rats. Random blood glucose was unchanged from initial hyperglycaemic values in the control group after 6 weeks, while PFJ rats had lowered their random blood glucose significantly (from 17·1 (sd 3·4) to 4·8 (sd 5·0) mmol/l). A reduced water intake was observed in experiments 3 and 4 compared with the chow-fed rats in experiments 1 and 2 due to the increased water content of these starch gel-based, semi-purified diets.

**Table 5.** Effect of palm fruit juice (PFJ)‡ added directly to a semi-purified moderate-carbohydrate (CHO) diet§ for 6 weeks on hyperglycaemia in 12-week-old male Nile rats (*Arvicanthis niloticus*) (experiment 4) (Mean values and standard deviations)‖

Group...	Moderate-CHO		Moderate-CHO + PFJ	
	Mean	sd	Mean	sd
Rats (*n*)	5		5	
BW (g)				
Initial at age 12 weeks	110	12	118	10
After 6 weeks	119	12	111	9
Food intake				
g/d, 0–2 weeks	8	1	5*	1
g/d, 4–6 weeks	10	3	7*	1
kJ/d, 0–2 weeks	159	21	96*	25
kJ/d, 4–6 weeks	192	46	142*	13
Water intake (ml/d)	21	19	16	8
GAE intake (mg/kg BW per d)	0		545	
Random blood glucose (mmol/l)				
Initial at 12 weeks of age	16·8	2·8	17·1	3·4
After 6 weeks	18·0	8·6	4·8*†	5·0

BW, body weight; GAE, gallic acid equivalents; ppm, parts per million.

* Mean value was significantly different from that of the moderate-CHO group (*P* < 0·05; unpaired *t* test).

† Mean value was significantly different from that at 12 weeks of age (*P* < 0·05; paired *t* test).

‡ 800 ml of PFJ 13 000 ppm GAE for final concentration of 10·4 g GAE/kg diet.

§ Percentage energy from CHO–fat–protein = 40:43:17, 18·8 kJ/g.

‖ Data normalised by log transformation for statistical analysis as necessary.

### Experiment 5: direct comparison between palm fruit juice in food or drink

#### Energy intake, growth and blood glucose

This 4-week study revealed that weanling rats consuming unsweetened PFJ added to their food at a rate of 5·4 g/kg diet consumed 20 % less energy and weighed significantly less than either the control rats or rats consuming PFJ as a drink (Table 6). Because the PFJ as a drink was also unsweetened (in contrast to experiments 1 and 2) the consumption of PFJ was curtailed by bitterness, but water intake was sufficient from the starch-gel diet to not limit food intake or growth. No differences in food efficiency were observed. Despite the lower energy intake in the PFJ-diet rats, terminal random blood glucose was equally reduced (*P* < 0·05) in both PFJ-diet and PFJ-drink intervention groups compared with control rats. However, the terminal fasting blood glucose did not differ across groups, as typically observed in early stages of high-CHO-induced diabetes^(^^15^^)^. Both the random and the fasting blood glucose values tended to be lowest in the PFJ-drink group, despite their lower GAE intake of PFJ polyphenols.

**Table 6.** Anti-diabetic effects of palm fruit juice (PFJ) both mixed into a high-carbohydrate (CHO) diet*† or provided as a drink‡ for 4 weeks in 3-week-old male Nile rats (*Arvicanthis niloticus*) (experiment 5) (Mean values and standard deviations)§

Group...	Control		PFJ diet		PFJ 1500 drink	
	Mean	sd	Mean	sd	Mean	sd
Rats (*n*)	8		8		7	
BW (g)						
Initial at age 3 weeks	37	7	35	8	35	6
After 4 weeks	77^a^	8	70^a^	10	74	5
Food intake						
g/d	8^a^	1	7^a,b^	1	8^b^	0
kJ/d	134^a^	25	117^a,b^	13	134^b^	8
Drink intake						
ml/d	18^a^	7	21^b^	7	10^a,b^	2
kJ/d	0		0		4	0
Total energy intake (kJ/d)	134^a^	25	117^a,b^	13	138^b^	8
Food efficiency (g BW gained/1000 kJ)	10·7	1·3	11·1	0·9	10·4	0·8
GAE intake (mg/kg BW per d)	0		720		273	
Random blood glucose (mmol/l)						
After 4 weeks	13·4^a,b^	7·4	7·1^a^	6·7	6·1^b^	3·3
Fasting blood glucose (mmol/l)						
After 4 weeks	4·3	2·1	3·9	1·2	3·2	0·8
Organ weight (% BW)						
Liver	3·6	0·6	3·6	0·5	3·3	0·2
Kidney	0·8	0·2	0·9	0·2	0·8	0·1
Caecum	1·4^a^	0·4	1·9^a^	0·6	1·6	0·5
Adipose						
Epididymal	2·9^a^	0·5	2·4^a^	0·8	3·0	0·8
Perirenal	1·4^a^	0·4	1·1^a,b^	0·4	1·5^b^	0·4
Brown	1·7^a^	0·2	1·5^a,b^	0·3	2·0^b^	0·5
Total adipose	6·0^a^	0·8	5·1^a,b^	1·1	6·5^b^	1·1
Carcass	73^a^	2	75	5	77^a^	5
Fasting plasma lipids, 4 weeks (mmol/l)						
TC	3·9	1·3	4·7	2·8	3·6	1·0
TAG	2·8^a^	1·3	1·9^a^	0·5	2·0	0·7
Fasting plasma insulin, 4 weeks (pmol/l)	0·6	0·3	0·6	0·4	0·6	0·2

BW, body weight; GAE, gallic acid equivalents; TC, total cholesterol; ppm, parts per million.

^a,b^ Mean values within a row sharing a common superscript were significantly different (*P* < 0·05; one-way ANOVA and Fisher's protected least significant difference (PLSD) test).

* Percentage energy from CHO–fat–protein = 70:10:20, 16·7 kJ/g.

† 415 ml of PFJ 13 000 ppm GAE for final concentration of 5·4 g GAE per kg diet.

‡ Drink of PFJ containing 1500 ppm GAE.

§ Data normalised by log transformation for statistical analysis as necessary.

#### Organ weights and plasma parameters

In keeping with their lower energy intake, rats in the PFJ-diet group had significantly lighter epididymal, perirenal and brown fat pads and reduced total body fat compared with both other groups (Table 6). Rats drinking PFJ had a significantly greater percentage of carcass weight. No difference was observed in plasma TC, but plasma TAG was significantly lower for rats eating the PFJ diet compared with the control group. Fasting insulin was similar and somewhat elevated across groups.

## Discussion

These experiments confirm the previously reported diabetogenic aspect of dietary CHO in the wild-type male Nile rat, fed either commercial chow or semi-purified diets^(^^13^^,^^15^^)^. In addition, the results from all five experiments demonstrate the anti-diabetogenic and otherwise metabolically beneficial effects of PFJ phenolics in this T2DM model. Varying circumstances, including differences in starting age, duration of study, types of diet and mode of PFJ delivery, were studied to develop a comprehensive overview of the effects of PFJ in a wide range of settings. An inherent genetic bias towards susceptibility or resistance to T2DM induced by CHO^(^^15^^)^ complicates interpretation (see below). Nonetheless, both glucose metabolism and lipid metabolism were positively affected by PFJ, with lower values being associated with normalised liver and kidney weights, which serve as organ markers of T2DM described previously^(^^13^^–^^15^^)^. Analysis and interpretation of results from all experiments provide answers to several related questions: what are the primary effects of PFJ in the Nile rat? Are the effects of PFJ related to the OPP content as measured by GAE? To what extent does dosage route (food *v.* drink) influence beneficial effects of PFJ? Is the effect of PFJ influenced by starting age, study length or composition of diet? Does PFJ treatment alter food and water intake or growth, and are there any toxic effects of PFJ?

### Primary effects

Since the wild-type Nile rats used in these studies typically display a wide genetic variability in gene expression related to diabetes susceptibility^(^^13^^,^^15^^)^, some parameters registered rather large standard deviations. This, however, is in accord with human data, where genetic variation is a key aspect of inter-individual differences that make an impact on lifestyle characteristics affecting T2DM outcome. These Nile rat responses thus represent a realistic aspect of the diet–gene interaction associated with T2DM and the metabolic syndrome in humans. Despite the variability, the main and consistent effect of PFJ in all studies was its ability to delay or mitigate the rise in blood glucose and lipids observed in both weanling and sexually mature control rats, or even to reduce pre-existing hyperglycaemia in the early stages of diabetes. The diabetes in non-PFJ-supplemented control rats was associated with secondary pathologies in liver and kidney identified by increased organ weights. In previous studies^(^^13^^,^^15^^)^ the increase in liver weight was linked to the accumulation of glycogen and TAG, not unlike the steatosis reported for humans developing the metabolic syndrome and T2DM^(^^18^^)^. In a similar fashion, enlarged kidneys were associated with the hyperglycaemia and polyuria that develop in the Nile rat consuming diabetogenic diets^(^^13^^,^^15^^)^. Increased blood urea N in diabetic rats also signalled functional kidney damage^(^^15^^)^.

### Effect of oil palm phenolic intake on hyperglycaemia

The graded PFJ intake in experiment 2 demonstrated an incremental protection by OPP against the rise in blood glucose that typically occurs in chow-fed Nile rats. An additional study (*n* 10–11; data not shown) revealed a modest but insignificant anti-hyperglycaemic effect of PFJ following an even lower OPP intake (91 mg GAE/kg body weight per d). Although rats consuming PFJ for 36 weeks in experiment 1 and 24 weeks in experiment 3 revealed elevated random blood glucose at the end of these respective experiments, their hyperglycaemia was significantly less than that of control rats, and fasting glucose of PFJ rats was in the normal range. Collectively these observations demonstrate that intake of OPP from PFJ deterred the development of hyperglycaemia observed in control rats, but the amount supplemented did not lead to complete long-term prevention of rising glucose in a few genetically prone rats after 24 and 36 weeks.

At least three mechanisms might explain the anti-diabetic effects of PFJ: (a) reduction in glucose absorption rate; (b) improved insulin sensitivity (decrease in insulin resistance); and/or (c) enhanced insulin secretion. The reduced insulin level for low OPP intake rats in experiment 2 suggests that the rate of glucose absorption was depressed or an insulin-sensitising effect of PFJ was in play. Greater OPP intakes in that experiment were associated with increased plasma insulin, suggesting possible enhancement of insulin secretion at higher doses. Synergistic effects of PFJ might thus contribute to the remarkably low terminal blood glucose in rats with high OPP intakes.

### Effect of palm fruit juice in different application modes

PFJ proved effective whether supplied as a drink or mixed into the diet. These two methods of application were directly compared in weanling rats in experiment 5, which revealed no difference in terms of blood glucose or fasting insulin. However, since the GAE intake of rats drinking PFJ was about one-third that of those with PFJ mixed into their diet, providing PFJ as a drink seems to be a more effective application method. Preliminary microarray studies using Illumina MouseRef-8 Version 2 Expression BeadChip (Illumina) indicated that the insulin signalling pathway, especially for phosphatidylinositol 3-kinase, was significantly down-regulated by PFJ^(^^19^^)^. Phosphatidylinositol 3-kinase has been reported to suppress glucose-stimulated insulin secretion^(^^20^^)^. PFJ seems to modulate this gene, which could at least partly explain an increased insulin secretion in rats given PFJ.

PFJ rats across experiments maintained significantly lower values for both random and fasting blood glucose. Positive results for PFJ also were observed for different starting ages, ranging from 3 weeks (weaning) to 12 weeks (young adults). In experiments 1 and 2 the PFJ protection persisted for as long as 24 weeks (experiment 2) and 36 weeks (experiment 1) of supplementation. This effectiveness, despite variations in age and diet (chow and semi-purified moderate-CHO or high-CHO diets), suggests that PFJ may be applicable as an anti-diabetic agent in different settings and population groups. The decrease in blood glucose observed in PFJ-supplemented, initially hyperglycaemic rats in experiment 4 raises the possibility that PFJ might serve not only as a preventive measure, but also as a treatment option during initial stages of diabetes. About 25 % of humans with resistance to insulin-stimulated glucose uptake by tissues do not progress to diabetes because insulin secretion remains sufficient to overcome the degree of insulin resistance^(^^21^^)^. Thus, the suggested reduction in glucose absorption or stimulation of insulin secretion by PFJ are potentially beneficial effects, if shown to pertain to humans.

### Effects of palm fruit juice on growth, and toxic effects

Unlike some anti-diabetic agents, PFJ did not affect growth (weight gain) in young adult Nile rats (experiments 1–4). This suggests that PFJ helped to maintain energy efficiency, a process that deteriorated as diabetes developed in control rats. The latter routinely become less energy efficient as they waste energy due to the inefficient metabolism of T2DM. By contrast, the 3-week-old weanling rats in experiment 5, given PFJ directly in the high-CHO diet, consumed less food (energy) and gained less weight (as adipose tissue) than the controls or rats given PFJ as a drink, which in turn drank less. This was the only experiment where the bitter taste of PFJ was not masked by Sucralose^TM^, which may account for the reduced food (PFJ in the diet) and drink (PFJ as drink) intakes. Reduced growth producing less diabetes is in accordance with our previous studies, where energy diluted by fibre or restricted to 75 % of *ad libitum* intake prevented diabetes in young male Nile rats^(^^13^^,^^15^^)^. The lack of any effect on food intake or weight in older rats given sweetened PFJ demonstrates that its positive influence did not depend on reductions in food consumption or body weight. The group receiving PFJ as a drink in experiment 5 even showed significantly greater carcass mass as a percentage of body weight, indicating better muscle growth for the same energy intake of control rats. Other than aversion to bitterness, no adverse effects were observed at any OPP intake, even at very high GAE intakes (600–720 mg/kg body weight in experiments 1, 2 and 5).

### Similarity of palm fruit juice to other natural extracts

A large body of research has described the beneficial metabolic effects of polyphenol-rich juices from various fruits or legumes in rodents and human clinical studies. For example, ginseng berry juice was found to lower blood glucose and body weight in *ob/ob* mice^(^^8^^)^, while an *Ichnocarpus frutescens* extract decreased blood glucose in rats with streptozotocin-induced diabetes^(^^22^^)^ and pomegranate peel and juice improved alloxan-induced diabetes in female rats^(^^9^^)^, as well as blood glucose levels and other parameters of the metabolic syndrome in human subjects^(^^23^^,^^24^^)^. Olive leaf extracts have improved glycaemic control in both rodent models and human subjects^(^^10^^)^, and cinnamon has demonstrated anti-hyperglycaemic properties in a number of studies^(^^25^^)^. Most recently, curcumin supplied to 240 pre-diabetics for 9 months at 1500 mg/d reportedly normalised their glucose metabolism^(^^12^^)^. Interestingly, both increases^(^^9^^,^^26^^)^ and decreases^(^^27^^)^ in plasma insulin levels have been observed in response to phenols. In summary, polyphenols from a variety of plant sources seem to exert a positive influence on both insulin sensitivity and secretion, both of which were observed in our Nile rats supplemented with PFJ.

Polyphenols also reportedly inhibit intestinal glucose absorption^(^^11^^,^^28^^)^, which is in keeping with certain data from Nile rats given PFJ (especially experiment 2, at 170 mg/kg body weight per d). Polyphenol-induced improvement of the metabolic syndrome, including lower blood pressure, has been reported in other studies, as well^(^^1^^,^^29^^)^. Thus, the beneficial influence of OPP from PFJ on blood glucose and plasma lipids and related metabolic parameters in Nile rats is consistent with numerous observations associated with natural plant extracts attributed to their polyphenol content. The inverse correlation between the dietary glycaemic index of a food and its content of polyphenols (i.e. more rapid glucose absorption with a lower amount of polyphenolics in the food) described in human subjects^(^^11^^)^ is in concert with the beneficial effect of PFJ supplementation protecting against deleterious high-CHO effects in the Nile rat.

*Post hoc* evaluation of dietary habits in Ghana showed that oil palm fruit is consumed by the local population on a regular basis, resulting in an OPP intake of about 300 mg/d, predominantly in soups and stews^(^^30^^)^. A preliminary clinical study comparing PFJ at 450 mg/d of OPP with a placebo for 4 weeks each in a cross-over design had no effect on a large number of metabolic parameters in twenty-five normoglycaemic, normolipaemic volunteers^(^^31^^)^.

### Conclusions

PFJ was shown to be an effective supplement to mitigate several aspects associated with hyperglycaemia in the male Nile rat. Its effects appeared to be relatively independent of starting age or application method (drink *v.* diet), although it seemed to be more effective if provided as a drink. OPP intake was positively correlated with an anti-hyperglycaemic effect. No impairment of energy intake or body-weight dynamics were observed in mature rats, nor were any other toxic effects attributed to PFJ. As such, PFJ has many of the characteristics of phenolic acids described for similar water-soluble extracts from fruits.
